# Macroalgal–bacterial interactions: identification and role of thallusin in morphogenesis of the seaweed *Ulva* (Chlorophyta)

**DOI:** 10.1093/jxb/eraa066

**Published:** 2020-02-04

**Authors:** Taghreed Alsufyani, Gianmaria Califano, Michael Deicke, Jan Grueneberg, Anne Weiss, Aschwin H Engelen, Michiel Kwantes, Jan Frieder Mohr, Johann F Ulrich, Thomas Wichard

**Affiliations:** 1 Institute for Inorganic and Analytical Chemistry, Friedrich Schiller University Jena, Jena, Germany; 2 Algal Research Laboratory, Chemistry Department, Science Faculty, Taif University, Taif, Saudi Arabia; 3 Jena School for Microbial Communication, Jena, Germany; 4 Centre for Marine Sciences (CCMAR), University of Algarve, Campus de Gambelas, Faro, Portugal; 5 University of Innsbruck, Austria

**Keywords:** Algal growth, cell wall, cross-kingdom interaction, morphogenesis, morphogenesis-promoting factor, phytohormone, rhizoid, seaweed, siderophore

## Abstract

Macroalgal microbiomes have core functions related to biofilm formation, growth, and morphogenesis of seaweeds. In particular, the growth and development of the sea lettuce *Ulva* spp. (Chlorophyta) depend on bacteria releasing morphogenetic compounds. Under axenic conditions, the macroalga *Ulva mutabilis* develops a callus-like phenotype with cell wall protrusions. However, co-culturing with *Roseovarius* sp. (MS2) and *Maribacter* sp. (MS6), which produce various stimulatory chemical mediators, completely recovers morphogenesis. This ecological reconstruction forms a tripartite community which can be further studied for its role in cross-kingdom interactions. Hence, our study sought to identify algal growth- and morphogenesis-promoting factors (AGMPFs) capable of phenocopying the activity of *Maribacter* spp. We performed bioassay-guided solid-phase extraction in water samples collected from *U. mutabilis* aquaculture systems. We uncovered novel ecophysiological functions of thallusin, a sesquiterpenoid morphogen, identified for the first time in algal aquaculture. Thallusin, released by *Maribacter* sp., induced rhizoid and cell wall formation at a concentration of 11 pmol l^−1^. We demonstrated that gametes acquired the iron complex of thallusin, thereby linking morphogenetic processes with intracellular iron homeostasis. Understanding macroalgae–bacteria interactions permits further elucidation of the evolution of multicellularity and cellular differentiation, and development of new applications in microbiome-mediated aquaculture systems.

## Teaser

Thallusin was isolated from sea lettuce aquacultures and assigned novel morphogenesis-inducing properties. Specifically, thallusin promoted rhizoidal zone and cell wall formation; thus, it plays crucial roles in cross-kingdom interactions.

## Introduction

The genus *Ulva* (Ulvales, Chlorophyta) comprises a group of green macroalgae which grows predominantly in intertidal zones. Eutrophication of coastal waters results in rapid growth of some macroalgal species, significantly increasing their biomass and thus forming, e.g. green tides (Fletcher, 1996; Smetacek and Zingone, 2013; Zhang *et al.*, 2019). *Ulva* species are characterized by either a tubular (‘enteromorpha’) or a flattened form (‘sea lettuces’) (Blomster *et al.*, 2002; Hayden *et al.*, 2003), but both morphotypes can also appear concomitantly in some species, such as *Ulva compressa* and *Ulva mutabilis* (Tan *et al.*, 1999; Steinhagen *et al*., 2019*a*, *b*). Remarkably, the growth, cell differentiation, and morphogenesis of *Ulva* species depend on their interaction with specifically associated bacteria and the chemical mediators these bacteria produce (Goecke *et al.*, 2010; Egan *et al.*, 2013; Wichard, 2015). Given that multicellularity evolved independently in the lineage preceding *Ulva* evolution (Coates *et al.*, 2014), it is interesting to decipher which molecular mechanism regulates morphogenesis and, in particular, the contribution of bacteria to this regulation process. Furthermore, the unraveling of these pathways may be facilitated by the rather simple construction of the vegetative thallus of *Ulva*, which consists of only three cell types: rhizoid, stem, and blade cells. Interestingly, the fast-growing and naturally occurring ribbon-shaped *U. mutabilis* mutant, ‘slender’, lacks stem cells and thus develops only primary rhizoids compared with the stronger holdfast of the flattened wild type (Spoerner *et al.*, 2012).

The activity of various algal growth- and morphogenesis-promoting factors (AGMPFs) released by bacteria has been previously determined in coastal waters and land-based algal aquaculture systems (Grueneberg *et al.*, 2016; Ghaderiardakani *et al.*, 2019). Accumulated data have suggested that microbial action may contribute plant hormone-like substances to natural waters (Matsuo *et al.*, 2005; Marshall *et al.*, 2006; Singh *et al.*, 2011; Spoerner *et al.*, 2012). It is thus possible that the coastal zone, which favors microbial growth due to land drainage, is rich in AGMPFs that are potentially associated with substantial economic implications (Provasoli, 1958). Identification of these chemical mediators has been under discussion for nearly 70 years, since the phycologist Provasoli stated, ‘To resolve these issues, [there] are not only extensive pure culture studies needed but also convenient sensitive methods for assaying plant hormones in seawater’ (Provasoli, 1958).

**Fig. 1.**
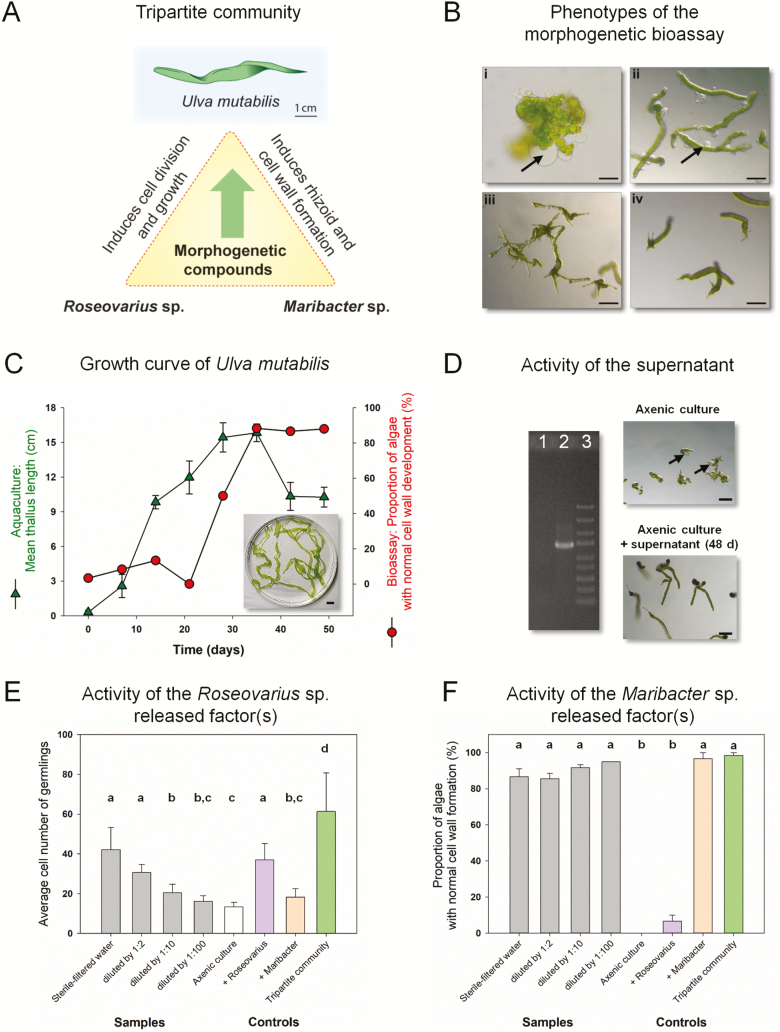
*Ulva mutabilis* as a model organism for bacteria-induced morphogenesis (A) *U. mutabilis* (‘slender’) and two essential bacteria which release morphogenetic factors establish a tripartite community. (B) The axenic callus-like morphotype of *U. mutabilis* was compared with axenic cultures (i) inoculated with bacteria of the *Roseovarius* sp. only (ii), bacteria of the *Maribacter* sp. only (iii), or both bacterial strains (iv). Arrows indicate the typical colorless protrusions from the exterior cell walls due to lack of morphogens released by *Maribacter* sp. Scale bar=500 µm (i); 100 µm (ii–iv); image (iii) with permission adapted from Wichard (2015). (C) Growth curve of *U. mutabilis* in a land-based tank system (error bars: ±SD, *n*=3). The morphogenetic activity of AGMPFs in sterile-filtered water samples is given as a portion of normally developed cell walls using the ‘*Ulva* bioassay array’ (inset: algae in stationary phase, scale bar=1 cm). (D) Demonstration of axenicity by PCR amplification of a part of the 16S rDNA gene extracted from the supernatant of the purified gamete stock solution (lane 1) and the non-axenic culture of *U. mutabilis* (lane 2). Lane 3 shows the GeneRuler DNA Express Ladder (Thermo Fisher Scientific) (scale bar=100 µm). (E, F) The AGMPFs in sterile-filtered water samples derived from aquacultures on day 48 were determined using two different bioassays. *Roseovarius*-released factor(s) were distinguished (E) from *Maribacter*-released factor(s) (F) by performing a dilution series. Data were analyzed by one-way ANOVA with Tukey’s post-hoc test (*P*<0.01, error bars: SE, *n*=60). Mean values accompanied by differing letters differed significantly. Axenic cultures (white) were inoculated with sterile-filtered water (gray) or *Roseovarius* sp. (purple), *Maribacter* sp. (orange), or both (green).

Given that the life cycle of *U. mutabilis* can be controlled entirely under laboratory conditions (Stratmann *et al.*, 1996), its gametes can develop parthenogenetically (Løvlie and Bryhni, 1978), and axenic cultures of this species are available (Spoerner *et al.*, 2012), the development of cell types can thus be tested through the application of an engineered microbiome (Wichard, 2015). Therefore, *U. mutabilis* has been utilized as a model organism for investigating the development of multicellularity and morphogenesis (Wichard *et al.*, 2015; De Clerck *et al.*, 2018) (Fig. 1A).

Under axenic conditions, *U. mutabilis* zoids or gametes develop into callus-like structures that appear as pincushion morphotypes characterized by atypical cell wall formation with protrusions, absence of cell differentiation and rhizoid formation, and slow growth (Spoerner *et al.*, 2012). Similarly, axenic gametes inoculated with *Roseobacter* clade bacteria develop into dark green distorted germlings; these propagules do not develop rhizoids and become entirely covered with protrusions (Fig. 1B) (Spoerner *et al.*, 2012; Ghaderiardakani *et al.*, 2017). Therefore, the phenotype of *U. mutabilis* associated with *Roseobacter* bacteria has often been incorrectly described as axenic. However, when *Roseovarius* sp. (MS2) and *Maribacter* sp. (MS6) were re-seeded with axenic gametes, forming a tripartite community, all growth and developmental deficiencies associated with the thallus were entirely abolished through the release of AGMPFs (Fig. 1A). Known phytohormones cannot replace these factors at naturally relevant concentrations (Spoerner *et al.*, 2012; De Clerck *et al.*, 2018). Currently, thallusin is the only identified algal morphogenesis inducer (morphogen) isolated from an epiphytic marine bacterium strain [YM2-23 (GenBank AB073558)], which belongs to the Cytophaga–Flavobacterium–Bacteroides group and was isolated from the green macroalga *Monostroma oxyspermum* (Ulvales, Chlorophyta) (Matsuo *et al.*, 2005). Other strains with similar morphogenetic activity were further clustered into a clade comprising *Cytophaga* sp. or *Zobellia uliginosa* (Matsuo *et al.*, 2003).

Matsuo *et al.* (2005) described the effect of thallusin at low concentrations (0.001–1 pg l^−1^) as a trigger for the development of the thallus in *M. oxyspermum.* In addition, they reported that thallusin partially promotes the formation of distromatic thalli in *Ulva pertusa*. However, the described effects deviated from our observations in axenic cultures of *U. mutabilis* or *Ulva intestinalis,* which require at least two bacteria with differing properties to affect their development and morphogenesis (Spoerner *et al.*, 2012; Ghaderiardakani *et al.*, 2017). In *Ulva*, AGMPFs can be categorized as follows: the *Roseovarius*-released factor resembles a cytokinin functions by promoting cell division, whereas the *Maribacter*-released factor acts similarly to auxins (e.g. indole-3-acetic acid) by promoting rhizoid initiation and cell wall formation (Spoerner *et al.*, 2012).

Herein we sought to identify AGMPFs, such as thallusin, located in the phycosphere of *Ulva* that are capable of phenocopying the activity of *Maribacter*-released factors in complementation assays with *Roseovarius* sp. (Fig. 1A, B: Supplementary Fig. S1 at *JXB* online). As Matsuo *et al.* (2005) previously identified thallusin-producing bacteria, localized to a subset of Bacteroidetes within a clade that includes Flavobacteria, we hypothesized that thallusin was the primary AGMPF released by *Maribacter* spp. into the chemosphere of *Ulva* spp.

Therefore, in this study, we attempted to determine the role of thallusin in the *U. mutabilis* (morphotype ‘slender’) tripartite community within aquacultures (Faro, Portugal). Overall, our approach demonstrated that ecologically reconstructed macroalgal–bacterial interactions can contribute to the current understanding of the evolution of cellular differentiation and multicellularity.

## Materials and methods

### Algal aquaculture

Haploid gametophytes from *Ulva mutabilis* Føyn (sl-G[mt+]; morphotype ‘slender’; *locus typicus*: Ria Formosa, Portugal) were used in all bioassays and aquacultures. The laboratory strains are direct descendants of the original isolates collected by B. Føyn on the south coast of Portugal in 1952 (Føyn, 1958; Løvlie, 1964). The isolate was recently phylogenetically reclassified and renamed *Ulva compressa* ‘mutabilis’ (Steinhagen *et al.*, 2019*a*). For isolation of AGMPFs, *U. mutabilis* aquaculture (V=200 liters) was conducted at the Ramalhete Station of the Centro de Ciências do Mar (CCMAR) in Faro (Portugal). Conical tanks, made of polyester resin reinforced with fiberglass, were used for cultivation to minimize the accumulation of leached chemicals that may interfere with MS. Before usage, all tanks were washed with a 10% HCl water solution (v/v) and subsequently with commercially available bleach. After complete removal of bleach by washing with pure water, tanks were filled with 200 liters of 10 µm filtered artificial seawater (33.2 g l^−1^ of Instant Ocean obtained from the Institute for Inorganic and Analytical Chemistry, Aquarium Systems, Sarrebough, France). Trace metals, Fe^III^-EDTA, and vitamins were added based on the final concentrations of the standardized *Ulva* culture medium (UCM) (Stratmann *et al.*, 1996; Califano and Wichard, 2018). Tanks were continuously aerated to avoid algal accumulation at the bottom, which would affect algal growth. Air was filtered through HEPA-Vent filters (Ø 50 mm, Whatman, Germany) and entered into the tanks from the bottom through a 6 mm diameter polyethylene hose culminating in a glass Pasteur pipette. All tanks were covered with Tygon film. For the starter cultures, axenic gametes were prepared and inoculated with the *Roseovarius* sp. strain MS2 and *Maribacter* sp. strain MS6. Germlings (0.5 cm length) were used for aquaculture inoculation (50 germlings l^−1^). Final optical density (OD_620_) of the *Roseovarius* sp. and *Maribacter* sp. was adapted to 0.0001.

Under standardized laboratory conditions, *U. mutabilis* was cultivated in UCM, applying a light/dark regime of 17/7 h with light intensity set at 60–120 µmol photons m^−2^ s^−1^ (Stratmann *et al.*, 1996; Califano and Wichard, 2018). To monitor the growth of *U. mutabilis* in the aquacultures, the length of the tubular thallus was measured in triplicate (Løvlie, 1964).

### Bacterial cultures

*Maribacter* sp. strain MS6 (GenBank EU359911) and *Roseovarius* sp. strain MS2 (GenBank EU359909) were cultivated in marine broth-enriched UCM (50%, w/v; Roth, Karlsruhe, Germany) for inoculation of *Ulva* cultures. To determine AGMPFs in the bacterial supernatant, *Maribacter* sp. strain MS6 was cultivated in UCM supplemented with marine broth (10%, w/v, Roth) in sterile Nalgene^®^ polycarbonate bottles (Thermo Fisher Scientific, Schwerte, Germany). A 10 ml aliquot of an exponentially growing *Maribacter* MS6 starter culture (OD_620_=1.0) was used for the inoculation of 10 liters of marine broth-supplemented medium. The culture was grown at 20–22 °C, stirred continuously, and supplied with sterile air through a HEPA-VENT-filter system (Whatman™; Thermo Fisher Scientific). Subsequently, the culture was harvested at OD_620_=1.0, centrifuged at 3000 *g*, and filtered through Whatman™ GF/F filters (Thermo Fisher Scientific).

### Preparation of axenic gametes and survey for morphogenetic activity in bioassays

Gametogenesis of mature *U. mutabilis* was triggered by mincing the thallus and draining of sporulation inhibitors from algal fragments (Stratmann *et al.*, 1996, Vesty *et al.*, 2015). Following the removal of a swarming inhibitor through an additional medium exchange, gametes were released 3 d later. Released gametes were purified from bacteria using sterile equipment in a laminar flow cabinet by exploiting the positive phototaxis of gametes. The stock solution of axenic gametes was diluted with UCM or the respective sterile-filtered aquaculture water to reach a final density of 20–40 settled gametes per well (200 μl) conducted in 96-well microarray plates (Grueneberg *et al.*, 2016). Axenicity of the purified gamete stock was confirmed via PCR using primers and cycling conditions previously described (Califano and Wichard, 2018). Following inoculation, gametes settled within 24 h in the darkness and were used in the ‘*Ulva* bioassay array’ (Grueneberg *et al.*, 2016).

### Determination of growth- and morphogenesis-promoting factors

To survey the prevalence of morphogenesis-inducing AGMPFs under controlled conditions, the ‘*Ulva* bioassay array’ was performed. All water samples derived from algal or bacterial cultures were passed twice through 0.2 μm polyethersulfone filters (Roth) and inoculated in 96-well plates containing axenic gametes. The bioassay array was performed with a dilution series of the sterile-filtered samples using UCM to avoid nutrient deficiency during the experiment. A dilution step refers to the total volume (*x*/[*x*+*y*]), where *x* indicates the parts of the collected aquaculture water, and *y* denotes the parts of UCM (Grueneberg *et al.*, 2016). In total, three biological replicates with 20 individuals each were carried out. Qualitative features, including the presence of longitudinal growth (number of cells), differentiated rhizoids, and appearance of cell walls with protrusions, were inspected under an inverted microscope. After 7–10 d, and upon the initial appearance of malformed cell walls in the negative (axenic) control, the average cell number of the growing germlings and the percentage of thalli with entirely normal cell walls were determined. The log-transformed data set passed normality (Shapiro–Wilk), and an equal variance test (Brown–Forsythe) prior to one-way ANOVA with Tukey’s post-hoc test was performed (Minitab 18, Additive GmbH, Friedrichsdorf, Germany; SigmaPlot 13, Systat Software, Erkrath, Germany).

### Preparation of extracts containing AGMPFs

Sterile-filtered supernatants of algal (V=10 liters) or bacterial cultures (V=1 liter) were loaded on a solid-phase cartridge (Sep-Pak C18 Plus Long Cartridge; Waters™, Manchester, UK) conditioned with 15 ml of methanol. By applying a stepwise elution gradient (25, 75, and 100% MeOH/H_2_O, v/v), 15 ml of each fraction was collected, evaporated under a nitrogen stream, and resuspended in 0.5 ml of UCM for utilization in the ‘*Ulva* bioassay array’. The procedure was repeated several times to process 50 liters of aquaculture medium or 10 liters of *Maribacter* sp. MS6 bacterial growth medium. The bioassay is based on complementation of the *Roseovarius* factor(s) within the tripartite community following replacement of the *Maribacter* sp. by the tested extract containing AGMPFs (Weiss *et al.*, 2017). The activity of the extracts was compared with the synthetic reference standard (±)-thallusin (Nishizawa *et al.*, 2007), and model compounds, such as picolinic acid and sclareol as negative control.

### Ultra-high performance liquid chromatography (UHPLC) coupled with electrospray ionization (ESI) high-resolution mass spectrometry (HRMS) measurements

UHPLC coupled with high-resolution Orbitrap MS was carried out using an UltiMate HPG-3400 RS binary pump (Thermo Fisher Scientific). The Kinetex^®^ C-18 RP (50×2.1 mm; 1.7 µm; Phenomenex, Aschaffenburg, Germany) chromatography column was maintained at 25 °C within the TCC-3200 column compartment. Eluent A contained water with 2% acetonitrile and 0.1% formic acid. Eluent B was acetonitrile with 0.1% formic acid. The gradient applied was as follows: initial condition, 0.2 min, 0% B; 8.0 min, 100% B; 8.0–9.1 min, 100% B; 9.1–10 min, 0% B. All measurements were performed with a constant flow rate of 0.4 ml min^−1^. Samples were injected via the WPS-3000 autosampler equipped with a 25 µl injection syringe under temperature-controlled conditions at 10 °C. Mass spectra were recorded using the Q Exactive™ hybrid quadrupole-Orbitrap mass spectrometer (Thermo Fisher Scientific) coupled to a heated ESI source. To reduce source contamination, solvent delay was set to 0.2 min. For analyzing thallusin, targeted selective ion monitoring (tSIM) in the positive ionization mode was used with the following instrument parameters: [M + H]^+^ (458.21±0.2 *m/z*); resolution (280 000); AGC target (5×10^4^); maximum IT (254 ms); acquisition time frame (5.0–6.0 min). A simultaneous full scan was set to *m*/*z*=100–600; resolution (70 000); AGC target (5×10^6^); and maximum IT (254 ms). Further, the sheath gas flow rate was set to 40; aux gas flow rate 15; sweep gas flow rate 0; discharge current (8.0 µA); capillary temperature (350 °C); S-lens RF level (33); and vaporizer temperature (360 °C). MS/MS measurements were conducted with 30 eV collision energy.

### Metal isotope-coded profiling (MICP)

Methanolic subsamples of solid-phase extracts derived from either bacterial or algal growth medium were used for analyzing the iron complexation ability of thallusin. The MICP approach enables the identification of metallophores in complex matrices by using the stable isotopes, ^54^Fe/^58^Fe (Deicke *et al.*, 2014). To ensure the stability of the iron–thallusin complexes, UHPLC-ESI-HRMS measurements were performed as described above, but using neutral eluents. Obtained chromatograms were screened for an artificial isotopic pattern with a mass difference of Δ3.994 *m/z* (Deicke *et al.*, 2014). Synthetic (±)-thallusin was used as a reference standard for the UHPLC-ESI-HRMS measurements (Nishizawa *et al.*, 2007).

### Iron uptake experiments in gametes

Gametogenesis was induced to obtain gametes for the uptake experiments. Gametes were discharged in iron-free UCM under sterile conditions. Gametes were counted by flow cytometry (Califano and Wichard, 2018), and subsequently mobile gametes were used directly. Aliquots of 2 ml of iron-free culture medium, each containing 3.5×10^7^ gametes, were transferred to 5 ml plastic tubes (Eppendorf, Hamburg, Germany), and ^58^FeCl_3_, ^58^Fe-EDTA, or ^58^Fe–thallusin was added to these tubes. Iron complexes were prepared in advance by mixing isotopically pure ^58^Fe as ferric chloride with a 10-fold excess of EDTA or thallusin, respectively. After 2 min and 10 min of continuous shaking, gametes were filtered (nitrocellulose filter, pore size 0.4 µm, Ø 25 mm, Sarstedt, Hildesheim, Germany) and washed with 15 ml of an aqueous solution containing Na-EDTA (0.05 mol l^−1^) and sodium oxalate (1 mol l^−1^), followed by a wash with 15 ml of an aqueous NaCl solution (0.45 mol l^−1^). Gametes were filtered and washed without the addition of iron species. Additional blanks (without gametes) were prepared by adding ^58^Fe and ^58^Fe-EDTA to the filters to correct for potential background absorbance. All treatments were conducted in triplicate. All filters were digested with 1 ml of ultrapure HNO_3_ (65%, v/v; Thermo Fisher Scientific) for 30 min at 70 °C. Clear solutions were diluted by transferring 150 µl to 4.35 ml of ultrapure water and 0.5 ml of yttrium solution (final concentration: 1 µg l^−1^). The ^58^Fe content was determined using an Agilent 7500c ICP-MS system (Agilent, Santa Clara, CA, USA) equipped with an ASX-500 autosampler (CETAC Technologies Inc., Omaha, NE, USA), a Babington nebulizer, a Scott spray chamber (cooled to 2 °C), and a Fassel torch (Agilent). For calibration, five solutions with varying concentrations of pure ^58^Fe in the range between 1 µg l^−1^ and 10 µg l^−1^ were prepared. Yttrium was measured as an internal standard.

## Results and discussion

To identify specific AGMPFs related to cell differentiation processes in *U. mutabilis*, we employed a functional complementation assay. Specifically, the assay performed was designed to assess the activity of solid-phase extracts from the supernatant of *Ulva* aquacultures and *Maribacter* sp. cultures. If the extract can exert morphogenetic activity, complementary to the *Roseovarius-*released factor, complete morphogenesis of *U. mutabilis* would be restored.

### Monitoring algal growth and morphogenesis-promoting factors during cultivation of *Ulva* mutabilis

Following inoculation with gametes, within 7 weeks, *U. mutabilis* developed normally in the presence of its microbiome in a closed aquaculture system with thalli of 15±1.8 cm in length. In addition, nutrients and AGMPFs were present in sufficient amounts. During the stationary phase, growth continued; however, large thalli broke into smaller parts (Fig. 1C).

The ‘*Ulva* bioassay array’ was employed to determine the morphogenetic activity of the sterile-filtered growth medium; we revealed that this medium induced complete morphogenesis recovery in *U. mutabilis* under axenic conditions (Fig. 1D). Hereby, it was further apparent that the morphogenetic activity of the sterile-filtered medium in aquaculture increased in parallel with the adult growth of *U. mutabilis* (Fig. 1C). During the early stages, when only weak morphogenetic activity was observed in the sterile-filtered water, algae developed as germlings, indicating the presence of a small amount of morphogens produced by very few bacteria. Subsequently, a rapid turnover of morphogenetic activity occurred, correlating with an increase in the growth of *U. mutabilis* and the depletion of the AGMPFs in the medium during this early period of cultivation (Fig. 1C).

Interestingly, following dilution of *Roseovarius-*released factor(s) below a concentration threshold of 1:100, the germling cell number was no longer distinguishable from that of axenic control cultures (Fig. 1E). Consequently, the *Maribacter-*released factor(s) solely controlled the development of *U. mutabilis* (Fig. 1F). Although no thallus was formed, rhizoid and cell wall developed normally. Further, the morphogenetic activity of the supernatant, even at a 100× dilution, did not significantly differ from that of the axenic gametes inoculated with the *Maribacter* sp. positive control (Fig. 1F).

Finally, the entire medium was harvested in the stationary phase, at which point the activity was found to be the highest, and examined for AGMPF activity as well as for purification of the *Maribacter*-released factor(s) (Fig. 1C). Solid-phase extraction and step gradient elution from up to 50 liters of aquaculture water resulted in enrichment of the factor that phenocopied *Maribacter* sp. activity by 10^6^, thus permitting MS analysis. The induction of rhizoid and cell wall formation activity was identified in the eluent ranging from 25% to 75% methanol (v/v), which did not contain *Roseobacter*-released morphogens; thus, these morphogens appeared to be more polar. These results confirmed earlier observations in coastal water and integrated multitrophic aquaculture systems (Grueneberg *et al.*, 2016; Ghaderiardakani *et al.*, 2019).

### Identification of thallusin and its iron complexes in the chemosphere of *Ulva* species

**Fig. 2.**
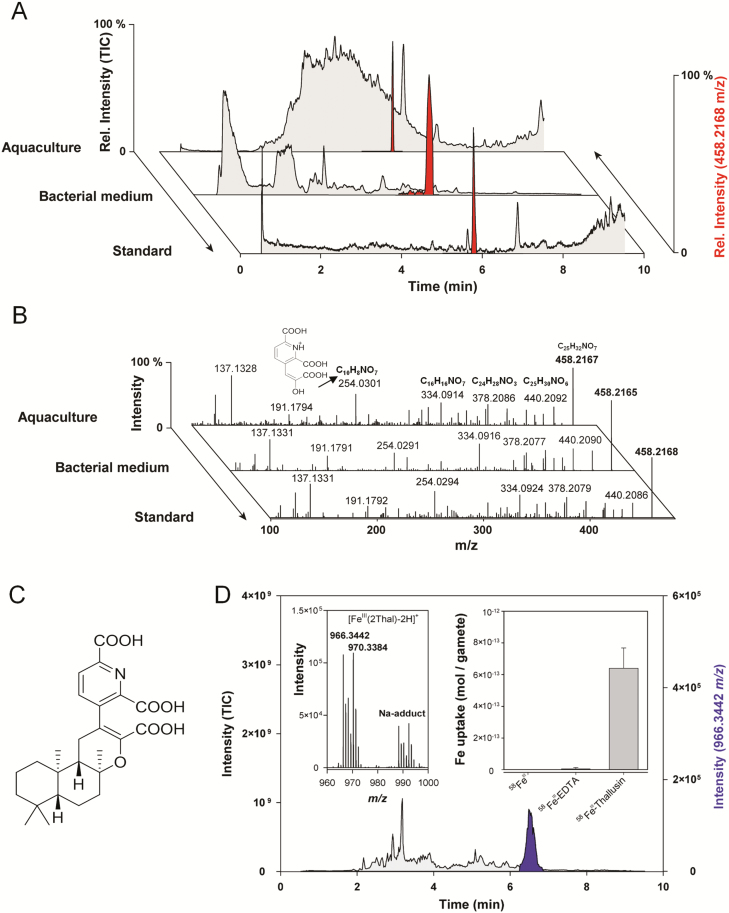
Identification of thallusin in the chemosphere of *Ulva mutabilis* (A) Algal morphogenesis-inducing fractions were analyzed by UHPLC-ESI-HRMS and compared with the reference standard of thallusin (red: extracted ion chromatograms of 458.2168 *m/z*). Samples were obtained from the supernatant of algal aquacultures and the bacterial growth medium of *Maribacter* sp. by solid-phase extraction. (B) MS/MS experiments of 458.2168 *m/z* indicate the substance identity of red-labeled peaks to coincide with that of (C) (–)-thallusin. Fragment 254.0301 *m/z* belongs to 2,6-dicarboxy-3-(2-carboxy-2-hydroxyvinyl)pyridine-1-ium. (D) The Fe complex of thallusin (966.3442 *m/z*) was identified in the extracted ion chromatogram (purple) using metal isotope-coded profiling. The artificial isotopic signature (^54^Fe/^58^Fe) of the complex is shown (inset, left). *U. mutabilis* gametes easily acquired Fe–thallusin compared with iron hydroxides and Fe-EDTA during the short-term uptake experiments (inset, right, error bars: SD).

Whereas *Roseovarius* sp. can be replaced by many Alpha- and Gamma-proteobacteria releasing unknown morphogens with similar functions (Grueneberg *et al.*, 2016), the specificity of the macroalgal–bacterial interactions is defined by few genera of the Bacteroidetes, such as the *Maribacter* species (Weiss *et al.*, 2017). The solid-phase extract of 50 liters of aquaculture water was analyzed by UHLPC coupled with ESI-HRMS, which did not indicate a prominent peak in the total ion current (Fig. 2A). However, the monitoring of select ions with 458.2167 *m/z* [M+H]^+^ identified the morphogen thallusin as the AGMPF in the chemosphere of *U. mutabilis*. Thallusin identification was subsequently confirmed by comparison with a synthesized racemate reference standard and MS/MS spectra identity (Fig. 2B, C). Thallusin production could be attributed to the *Maribacter* sp., as it was also identified in the bacterial growth medium (Fig. 2A). However, it was not obtained from the medium used for *Roseovarius* sp. growth. Subsequently, thallusin was found in all examined *Maribacter* and *Zobellia* cultures (Supplementary Fig. S2), except for *Maribacter polysiphoniae*, in accordance with its lack of morphogenesis-inducing activity reported previously by Weiss *et al.* (2017).

Thallusin harbors free carboxylic residues and picolinic acid moieties that may support iron complexation and contribute to iron acquisition. Organisms often release low molecular weight molecules (i.e. siderophores), even in the chemosphere of *Ulva* spp. Wichard (2016), to recruit iron under limiting conditions. Thus, we performed MICP using pairs of ^54^Fe and ^58^Fe to identify the Fe^III^–thallusin complex [Fe^III^(2Thal-2H^+^)]^+^ in the obtained mass spectrum of the analyzed extracts derived from *U. mutabilis* cultures (Fig. 2D, inset). Moreover, short-term uptake experiments indicated that gametes effectively incorporated iron through thallusin. However, they were unable to acquire it from Fe^III^-EDTA or free Fe^III^ (Fig. 2D, inset), which forms oxyhydroxids at pH>3 and is mostly unavailable in the marine environment (Butler, 1998). In addition to iron acquisition, it was previously demonstrated that siderophores can regulate gene expression in bacteria (Mettrick *et al.*, 2009). Hence, gene expression analyses in future studies of *Ulva* spp. may serve to elucidate whether thallusin mediates morphogenesis and iron homeostasis, which is essential for many iron-dependent metabolic processes in algae such as photosynthesis (Geider *et al*, 1993).

### Thallusin promoted rhizoid and cell wall formation in *Ulva* spp.

Although the previously identified thallusin-producing strain YM2-23 (Matsuo *et al.*, 2003, 2005) has been reported to be closely related to *Maribacter* species (Weiss *et al.*, 2017), its ecophysiological functions appear to vary in studied biological systems. Therefore, we tested the hypothesis that the multiple functions of thallusin induced the observed phenomena depending on the recipient’s underlying mechanisms.

**Fig. 3.**
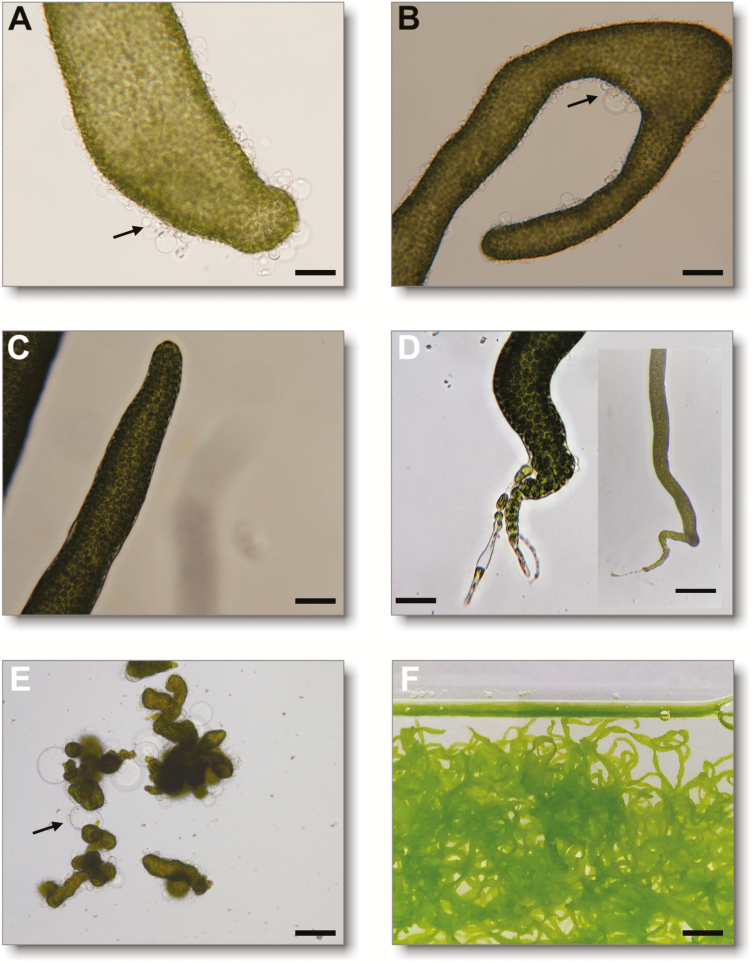
Thallusin complements the activity of *Roseovarius* sp. Axenic gametes of *Ulva mutabilis* were inoculated with the bacteria, *Roseovarius* sp., alone (A, B), or with thallusin (1.1×10^−10^ mol l^−1^; C, D). Arrows indicate the colorless protrusions from the exterior cell walls in the absence of thallusin. While no apical or basal end was recognized in the presence of *Roseovarius* sp. only, both the apical end (C) and the rhizoid (D) were clearly developed in the presence of thallusin. Axenic gametes developed into a callus [E, negative control; A–E, scale bar=500 µm; 200 µm (inset)]. In the presence of *Roseovarius* sp. and thallusin, *U. mutabilis* developed into the adult algae (scale bar=1 cm) (F).

Indeed, application of exogenous thallusin to axenic gametes induced the development of basal rhizoids and healthy cell wall formation (Fig. 3; Supplementary Fig. S3). Further, control experiments with *Roseovarius* sp. exhibited only typical cell divisions (Fig. 3A, B), whereas thallusin was essential to complete morphogenesis of *U. mutabilis* (Fig. 3C, D). Thallusin also stimulated protrusion elongation at the tip, causing separation from the stem cell by a septum and forming stretched primary rhizoid cells (Fig. 3D, inset). Rhizoids are important protuberances extending from the lower epidermal cells which establish the rhizoidal zone in *Ulva* spp. They similarly function as root hairs of vascular land plants by attaching algae to hard substrates. Importantly, indole-3-acetic acid and other examined phytohormones (zeatin, abscisic acid, gibberellins, and jasmonic acid) did not affect the development of germlings under our standardized conditions (De Clerck *et al.*, 2018). Given that the primary rhizoid of ‘slender’ individuals was not strong, growing algae detached from the substrate and floated in the aquaculture water (Fig. 1C), even under controlled conditions in the UCM after 2 weeks (Fig. 3F). Indeed, it was previously reported that a weak primary rhizoid characterizes the ‘slender’ morphotype (Løvlie, 1964).

We observed that the lowest effective concentration of thallusin was 1.1×10^–11^ mol l^−1^ (Supplementary Fig. S3), considering that (–)-thallusin has, thus far, proven to be the only biologically active enantiomer identified for *M. oxyspermum* (Gao *et al.*, 2006; Yamamoto *et al.*, 2014). Sclareol and picolinic acid, which are characterized by typical structural moieties similar to thallusin, exhibited no activity, and thus functioned as negative controls (Supplementary Fig. S4). It is noteworthy that thallusin previously triggered thallus formation (‘thallusin’) in *M. oxyspermum* (Matsuo *et al.*, 2005; Yamamoto *et al.*, 2014, 2018), whereas the same compound promoted cell wall and rhizoid formation (‘rhizoin’) in *U. mutabilis* in the current study. Taken together, these results indicate that thallusin will promote algal growth through healthy cell wall formation in *Ulva*, even in those algae which are drifting in aquacultures or coastal waters. Therefore, we concluded that thallusin functions as an essential chemical mediator for algal development; however, like plant hormones, thallusin possesses distinct functions in algal development depending on the receiver, and works synergistically with unknown *Roseovarius*-released factors as an algal morphogenesis inducer in *Ulva* spp. Moreover, thallusin seemed to be essential throughout the life cycle of *Ulva*, as protrusions of the cell wall appeared again in fast-growing algal cultures, indicating the depletion of the morphogen in the absence of *Maribacter* sp.

### Cross-kingdom interactions: setting up symbiosis through morphogens

**Fig. 4.**
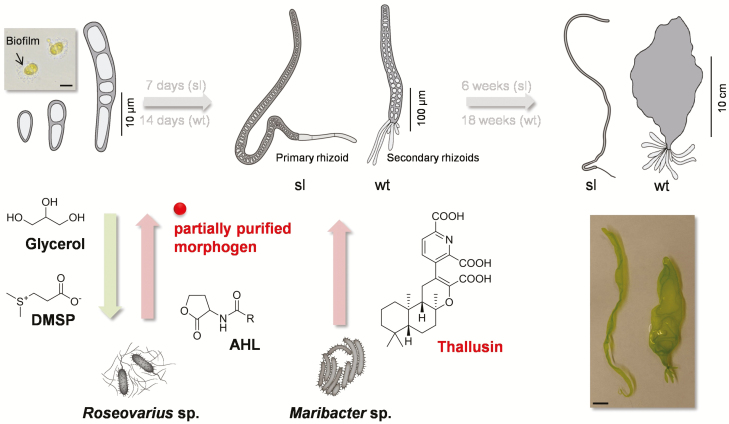
Chemical mediators in cross-kingdom interactions of *Ulva mutabilis* and associated bacteria. *Ulva mutabilis* provide a glycerol boundary layer as a carbon source for *Roseovarius* sp., thus supporting biofilm formation (Alsufyani *et al.*, 2017). *U. mutabilis* releases DMSP to attract *Roseovarius* sp. (Kessler *et al.*, 2018), which then promote cell division and growth through the secretion of unknown and partially purified morphogens [*Roseovarius*-released factor(s), red circle] (Spoerner *et al.*, 2012). *N*-Acyl homoserine lactone (AHL)-producing bacteria of the *Roseobacter* clade can also attract zoids of *U. mutabilis* through quorum-sensing signals (Joint *et al.*, 2002). AHLs differ in the length of their R-group side chain. Following completion of the first interactions, thallusin from *Maribacter* sp. promotes rhizoid and cell wall formation, thus finalizing the formation of the tripartite community. Top image: germling with bacteria (scale bar=10 µm). Bottom image: both morphotypes ‘slender (sl)’ and ‘wild type (wt)’ are shown (scale bar=1 cm).

To explore the mechanisms associated with macroalgae–microbe communication and to demonstrate that thallusin could form an essential endogenous morphogen, analytical chemistry was combined with specific bioassays. The observed interaction represents a further example of a more general ecological phenomenon in which bacteria are attracted to general signals and promote algal growth or morphogenesis in the phycosphere following recruitment (Amin *et al.*, 2015; Seymour *et al.*, 2017). In summary, the development of *Ulva* spp. is initiated by the settlement of mobile germ cells and resorbing of their flagella (Løvlie, 1964). We previously reported that *Roseovarius* sp. is chemotactically attracted to dimethylsulfoniopropionate (DMSP) and rapidly incorporates the metabolite (Fig. 4), although DMSP does not promote bacterial growth (Kessler *et al.*, 2018). However, *U. mutabilis* provides glycerol as a carbon source for *Roseovarius* sp., thus supporting biofilm formation. Additionally, bacteria have been reported to attract zoids via secretion of *N*-acyl homoserine lactones (Joint *et al.*, 2002; Tait *et al.*, 2005; Callow and Callow, 2011).

Following establishment of these initial interactions, an unknown morphogenetic compound stimulates cell divisions in *U. mutabilis* (*Roseovarius* factor) which promotes additional glycerol production (Kessler *et al.*, 2018), whereas thallusin (*Maribacter* factor) enables further mutualistic interaction through the formation of the rhizoidal zone and immobilization of the algae. Thus, thallusin-mediated rhizoid formation is of particular importance. Our results, in conjunction with those of Løvlie (1964), have revealed that natural development of the ‘slender’ mutant permits formation of only one (in some cases up to three) primary rhizoid. In this study, ‘slender’ was preferentially investigated given its fast growth and simplicity. Primary rhizoid cells are formed in the first stage of germling development following asymmetrical division of zoids, gametes, or zygotes. At the 10-cell stage, lateral protuberances of stem cells, located next to the primary rhizoid, are formed, leading to secondary and additional rhizoids which only develop in the wild type (Fig. 4; Løvlie, 1964). The tubular primary and secondary rhizoid cells predominantly grow unidirectionally, similar to a fungal mycelium (Spoerner *et al.*, 2012). In any case, *Maribacter* sp. induces the specific rhizoid formation of both morphotypes, ‘slender’ and wild type, according to their intrinsic program, as demonstrated by previous experiments (Spoerner *et al.*, 2012; Vesty *et al.*, 2015). Further transcriptomics studies will reveal the mechanisms underlying thallusin orchestration of rhizoid development, resulting in a primary rhizoid (‘slender’) or a rhizoidal zone (wild type).

In conclusion, thallusin acts as an essential hormone-like compound for algal growth and development by controlling the differentiation and cell wall formation of rhizoid and blade cells and triggering rhizoidal zone formation in *U. mutabilis*. This finding has important implications for advancing our current understanding of mutualistic interactions; further, they could enable successful deciphering of regulatory mechanisms of multicellularity-related genes via the activity of exogenous factors that have contributed to evolution in the green algae lineage. Further elucidation of these mechanisms is critical, especially given that the transition from unicellular to multicellular organisms consisting of different cell types was one of the greatest achievements in the evolution of complex life forms (Pires and Dolan, 2012). Therefore, understanding of the molecular and genetic processes triggered by thallusin will probably provide novel insights into the evolution of multicellularity.

## Supplementary data

Fig. S1. Cultivation of axenic *Ulva mutabilis* alone and in the presence of *Roseovarius* sp. or *Maribacter* sp. and *Roseovarius* sp. microbiome (tripartite community).

Fig. S2. Phylogenetic tree based on 16S rRNA gene sequences of *Maribacter* and relative strains.

Fig. S3. Axenic gametes, cultivated in *Ulva mutabilis* culture medium, spiked with thallusin.

Fig. S4. *Ulva mutabilis* morphogenesis bioassay of thallusin precursor molecules.

eraa066_suppl_supplementary_figures_S1_S4

## Abbreviations:

AGMPF: algal growth and morphogenesis-promoting factor
DMSP: dimethylsulfoniopropionate
MICP: metal isotope-coded profiling
UCM: *Ulva* culture medium
UHPLC: ultra-high performance liquid chromatography
