# Supplementary material for: Macroalgal–bacterial interactions: identification and role of thallusin in morphogenesis of the seaweed Ulva (Chlorophyta)
Source: J Exp Bot. 2020 Feb 4;71(11):3340–9. doi: 10.1093/jxb/eraa066 (PMC7289720; doi:10.1093/jxb/eraa066)
Supplement: eraa066_suppl_supplementary_figures_S1_S4 [file eraa066_suppl_supplementary_figures_s1_s4.pdf]

Supplementary data for

Macroalgal-bacterial interactions: Identification and role of thallusin in morphogenesis of the seaweed *Ulva* (Chlorophyta)

Taghreed Alsufyani[‡1,2], Gianmaria Califano[‡1], Michael Deicke[‡1], Jan Grueneberg[‡1,3], Anne Weiss[‡1,3], Aschwin H. Engelen[4], Michiel Kwantes[1], Jan Frieder Mohr[1,3], Johann F. Ulrich[1], Thomas Wichard [*,1,3]

[1]Institute for Inorganic and Analytical Chemistry, Friedrich Schiller University Jena, Lessingstr. 8, 07743 Jena, Germany

[2]Algal Research Laboratory, Chemistry Department, Science Faculty, Taif University, Taif 26571, Saudi Arabia

[3]Jena School for Microbial Communication, 07743 Jena, Germany

[4]Centre for Marine Sciences (CCMAR), University of Algarve, Campus de Gambelas, 8005-139 Faro, Portugal

[*]To whom correspondence should be addressed: thomas.wichard@uni-jena.de

[‡]Authors contributed equally to the study.

Content

Fig. S1. Cultivation of (A) axenic *Ulva mutabilis* alone and in the presence of (B) *Roseovarius* sp. or (C) *Maribacter* sp. and *Roseovarius* sp. microbiome (tripartite community).

Fig. S2. Phylogenetic tree based on 16S rRNA gene sequences of *Maribacter* strains.

Fig. S3. Axenic gametes, cultivated in *Ulva mutabilis* culture medium, spiked with thallusin.

Fig. S4. *Ulva mutabilis* morphogenesis bioassay of model molecules.

Figure S1

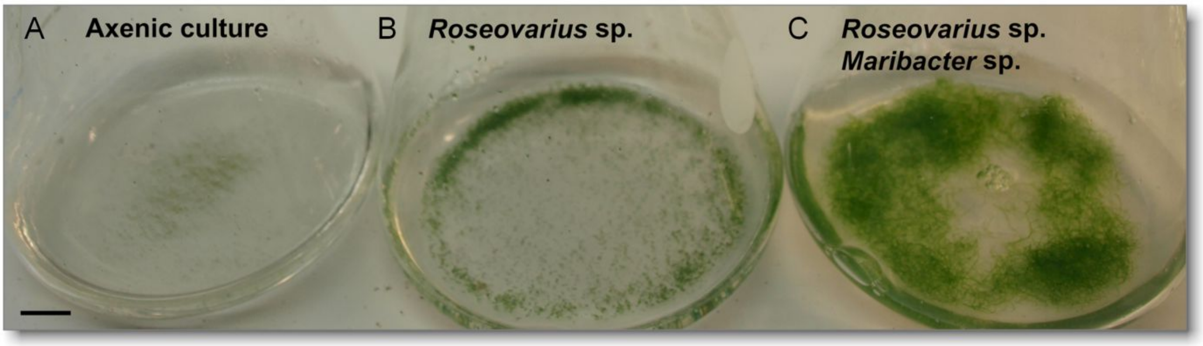

Fig. S1. Cultivation of (A) axenic *Ulva mutabilis* alone and in the presence of (B) *Roseovarius* sp. or (C) *Maribacter* sp. and *Roseovarius* sp. microbiome (tripartite community).

Figure S2

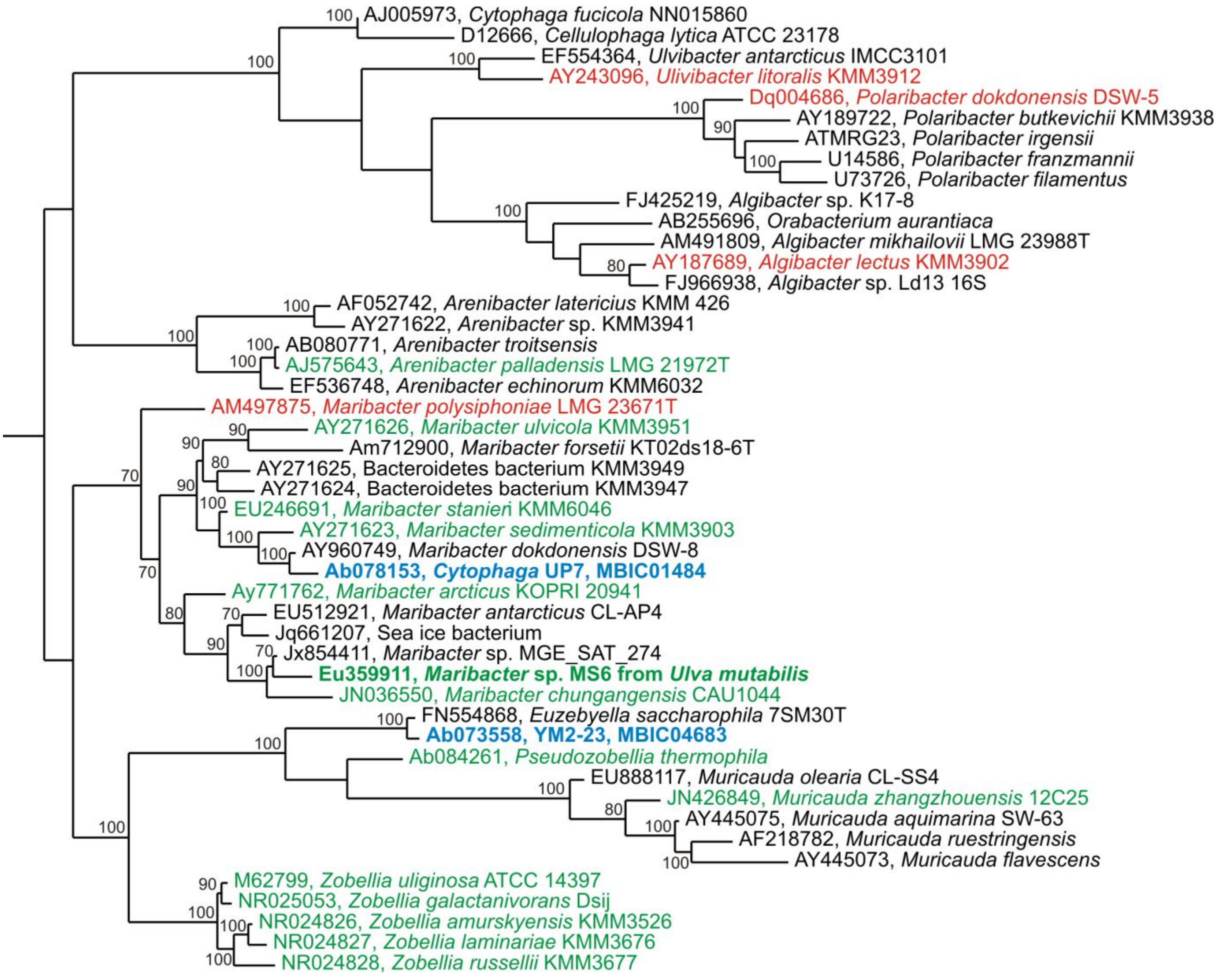

Fig. S2. Phylogenetic tree based on 16S rRNA gene sequences of *Maribacter* strains, including the *Maribacter* sp. MS6 strain, and representative members of related genera in the family Flavobacteriaceae, generated using the Maximum Likelihood phylogenetic tree. Numbers at nodes indicate bootstrap percentages (based on 500 bootstrap resamplings of data) when > 70 %. Thallusin producers are labeled in green and tested non-producers in red. Species in blue were described by Matsuo et al. (2003). The phylogenetic tree was adapted from Weiss *et al.* 2017.

Figure S3

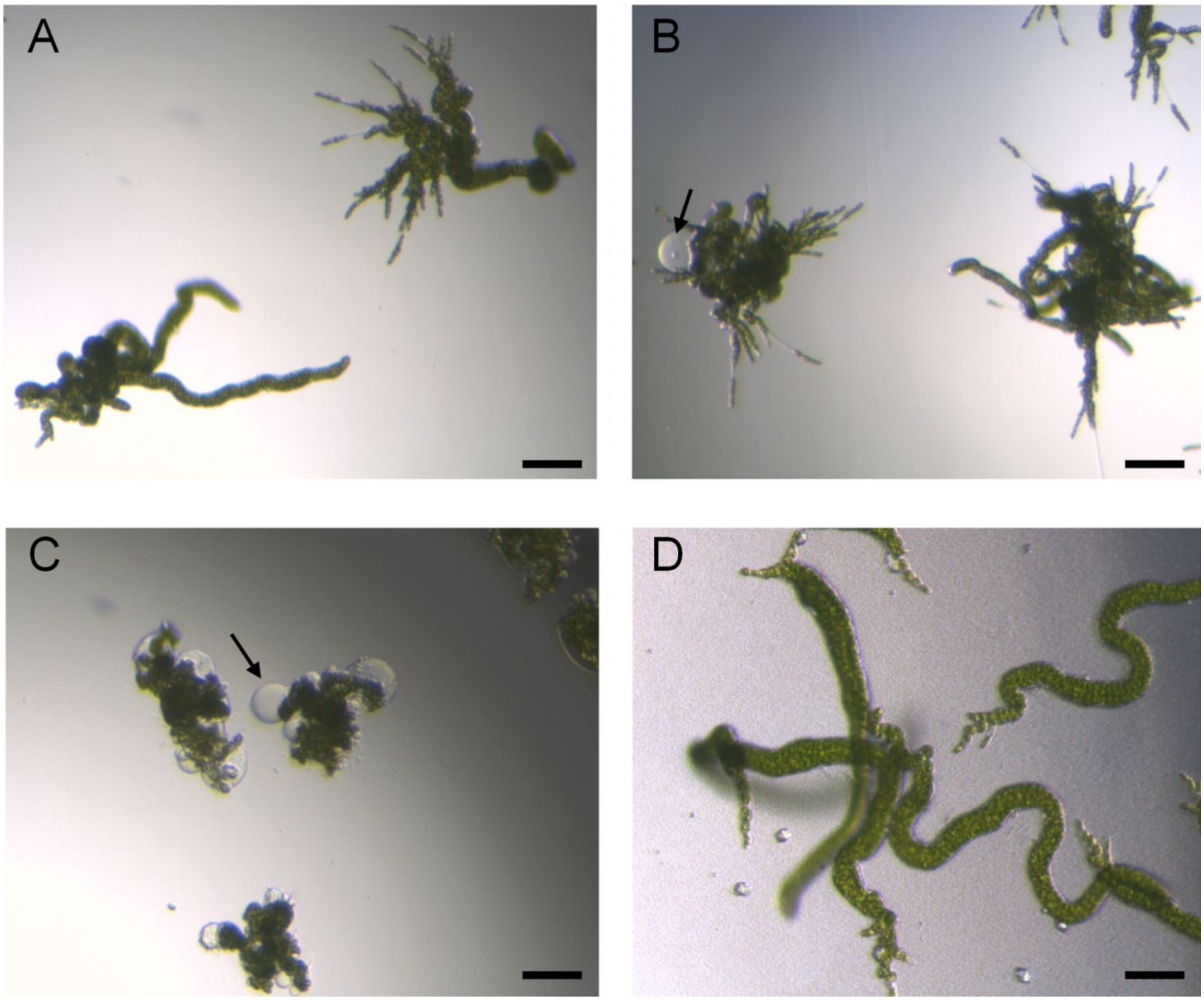

Fig. S3. Axenic gametes, cultivated in *Ulva mutabilis* culture medium, spiked with thallusin at a concentration of (**A**) 1.1 × 10^{-9} mol L^{-1} or (**B**) 1.1 × 10^{-11} mol L^{-1} (lowest observed effective concentration of rhizoid formation). (**C**) Axenic culture developed into undifferentiated calli (negative control). (**D**) Combination of thallusin (1.1 × 10^{-9} mol L^{-1}) and *Roseovarius* sp. recovers growth and morphogenesis of germlings. Scale bar: 100 µm. Three-week-old cultures of *U. mutabilis* are shown. Arrows point to protrusions of the cell wall, indicating a lack of thallusin.

Figure S4

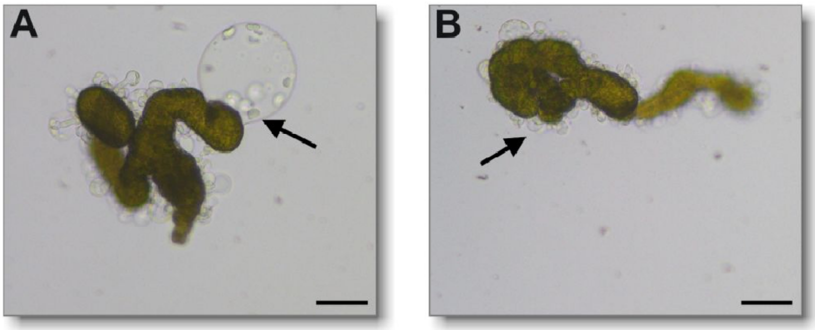

Fig. S4. *Ulva mutabilis* morphogenesis bioassay of model molecules (1×10^{-6} mol L^{-1}). (**A**) picolinic acid and (**B**) sclareol did not show morphogenetic activity when added to axenic cultures. Morphotypes did not differ from those of axenic cultures (Fig. 3E). Images show 3-weeks-old *U. mutabilis* germlings. Scale bar: 100 µm.
